# Late gadolinium-enhancement cardiovascular magnetic resonance imaging and angiographic characteristics of patients referred for evaluation of new onset cardiomyopathy

**DOI:** 10.1186/1532-429X-13-S1-P94

**Published:** 2011-02-02

**Authors:** Tatyana Danilov, Andrea Mignatti, Ruth P Lim, Leon Axel, Monvadi B Srichai

**Affiliations:** 1 New York Univeristy Medical Center, New York, NY, USA

## Background

Late gadolinium enhancement cardiac magnetic resonance (LGE-CMR) is an emerging non invasive tool for the assessment of etiology of new onset cardiomyopathy (CMP). Specific patterns of LGE-CMR have been shown to be accurate for distinguishing ischemic from non-ischemic CMP. We evaluated imaging and angiographic characteristics for different types of CMP based on LGE-CMR.

## Methods

Eighty nine consecutive patients were referred for evaluation of CMP by LGE-CMR. Patients were classified as ischemic CMP based on presence of subendocardial and/or transmural LGE in a coronary artery distribution pattern on CMR. Patients with none, midwall or subepicardial LGE pattern were classified as non ischemic CMP. Patients with mixed patterns of LGE (ie: subendocardial and midwall) were classified as mixed CMP. In the subset of 57 patients with recent angiography, the predictive value of CMR for diagnosing ischemic CMP (left main, proximal LAD, or 2 vessel disease) was assessed.

## Results

See Table 1.

**Table 1:** 

Imaging Characteristics	Ischemic CMP (62/89) 69.7%	Non ischemic CMP (18/89) 20.2%	Mixed CMP (9/89) 10.1%	P value
Male (%)	45/62 (72.6%)	9/18 (50%)	2/9 (22.2%)	0.16
*Age (years)*	59.9	59.4	58.8	NS
				
Left ventricular ejection fraction (%)	31.2	24.1	26.7	0.03
Left ventricular end-diastolic volume (ml)	227.8	267.1	236.8	0.20
Left ventricular end-systolic volume (ml)	162.4	210.8	185.0	0.07
Right ventricular dilatation (%)	8/62 (12.9%)	8/18 (44.4%)	2/9 (22.2%)	0.10
Mitral regurgitation (moderate to severe)	13/62 (21.0%)	10/18 (55.6%)	2/9 (22.2%)	0.004
Left ventricular thrombus	8/62 (12.9%)	0/18 (2%)	2/9 (22.2%)	0.17
Abnormal first pass perfusion	47/55 (85%)	6/15 (40.0%)	4/5 (80.0%)	0.0005
Average % myocardium with LGE	434/1054 (41.2%)	33/306 (10.8%)	54/153 (35.3%)	
LGE in LAD territory	23/62 (37.1%)	1/18 (5.6%)	4/9 (11.1%)	
LGE in LCX territory	4/62 (6.5%)	1/18 (5.6%)	2 (22.2%)	
LGE in RCA territory	12/62 (19.4%)	0/18 (0%)	4 (44.4)	
2 vessel territory	12/62 (19.4%)	1/18 (5.6%)	3/9 (33.3%)	
3 vessel territory	9/62 (14.5%)	3/18 (16.7%)	1 (11.1%)	

In the subgroup of 57 patients with recent angiography, CMR demonstrated 96% sensitivity, 63% specificity, and 91% diagnostic accuracy for distinguishing ischemic versus non-ischemic CMP. The positive predictive and negative predictive values were 94% and 71% respectively.

## Conclusions

The majority of patients referred for evaluation of CMP by LGE-CMR had ischemic CMP (73%), while 20% had a non ischemic etiology and 10% had a mixed CMP based on LGE characteristics. Non-ischemic and mixed CMP demonstrate significantly decreased function with trend towards increased left ventricular size despite smaller degrees of myocardial fibrosis. LGE-CMR has high diagnostic accuracy for the diagnosis of ischemic CMP.

